# Treadmill Pre-Training Ameliorates Brain Edema in Ischemic Stroke via Down-Regulation of Aquaporin-4: An MRI Study in Rats

**DOI:** 10.1371/journal.pone.0084602

**Published:** 2014-01-09

**Authors:** Zhijie He, Xiaolou Wang, Yi Wu, Jie Jia, Yongshan Hu, Xiaojiao Yang, Jianqi Li, Mingxia Fan, Li Zhang, Jinchun Guo, Mason C. P. Leung

**Affiliations:** 1 Department of Rehabilitation, Huashan Hospital, Fudan University, Shanghai, China; 2 Shanghai Key Laboratory of Magnetic Resonance, Department of Physics, East China Normal University, Shanghai, China; 3 State Key Laboratory of Medical Neurobiology, Shanghai Medical College, Fudan University, Shanghai, China; 4 Department of Rehabilitation Sciences, The Hong Kong Polytechnic University, Hung Hom, Kowloon, Hong Kong, SAR, China; National University of Singapore, Singapore

## Abstract

**Objective:**

Treadmill pre-training can ameliorate blood brain barrier (BBB) dysfunction in ischemia-reperfusion injury, however, its role in ischemic brain edema remains unclear. This study assessed the neuroprotective effects induced by treadmill pre-training, particularly on brain edema in transient middle cerebral artery occluded model.

**Methods:**

Transient middle cerebral artery occlusion to induce stroke was performed on rats after 2 weeks of treadmill pre-training. Magnetic resonance imaging (MRI) was used to evaluate the dynamic impairment of cerebral edema after ischemia-reperfusion injury. In addition, measurements of wet and dry brain weight, Evans Blue assay and Garcia scores were performed to investigate the cerebral water content, BBB permeability and neurologic deficit, respectively. Moreover, during ischemia-reperfusion injury, the expression of Aquaporin 4 (AQP4) was detected using immunofluorescence and Western bloting analyses.

**Results:**

Treadmill pre-training improved the relative apparent diffusion coefficient (rADC) loss in the ipsilateral cortex and striatum at 1 hour and 2.5 hours after cerebral ischemia. In the treadmill pre-training group, T2W1 values of the ipsilateral cortex and striatum increased less at 7.5 hours, 1 day, and 2 days after stroke while the brain water content decreased at 2 days after ischemia. Regarding the BBB permeability, the semi-quantitative amount of contrast agent leakage of treadmill pre-training group significantly decreased. Less Evans Blue exudation was also observed in treadmill pre-training group at 2 days after stroke. In addition, treadmill pre-training mitigated the Garcia score deficits at 2 days after stroke. Immunofluorescence staining and Western blotting results showed a significant decrease in the expression of AQP4 after treadmill ischemia following pre-training.

**Conclusions:**

Treadmill pre-training may reduce cerebral edema and BBB dysfunction during cerebral ischemia/reperfusion injury via the down-regulation of AQP4.

## Introduction

Ischemic stroke exhibits characteristics of higher morbidity, mortality and disability. Early thrombolytic therapy plays an important role in clinical management, but it also has been limited because of a narrow time window. The development of an effective and preventive treatment for stroke becomes an attractive topic of interest.

Treadmill training has been reported to induce brain ischemic tolerance via a reduction in inflammatory responses [1], increase in blood capillary [2] and improvement of blood brain barrier function as well [3]. Our previous studies have also shown that treadmill training can reduce the concentration of extracellular fluid glutamate and inhibit the expression of glutamate receptor after cerebral ischemia [4], [5], [6], [7]. Moreover, it has been shown that pre-training reduces brain water content after ischemia using wet and dry weight methods, and it was suggested that exercise pre-training could decrease brain edema. However, we cannot investigate this result in living animals via the this procedure [8]. Magnetic resonance imaging (MRI) has the advantage of enabling live dynamic observations compared to other methods.. Using 3T MRI, Pillai et al. [9] observed the biphasic nature of blood brain barrier(BBB) opening and the process of brain edema in a focal cerebral ischemia model [10].

For ischemic brain edema, there are two major types of brain edema: cytotoxic and vasogenic [11]. Cytotoxic edema results from the subtle disturbance in BBB permeability, which is associated with cellular disruptions in ionic homeostasis. The main feature of cytotoxic edema is the swelling of brain cells, in particular, the enlargement of astrocytic endfeet. Diffusion-weighted imaging (DWI), as a sequence of MRI can be used to detect cytotoxic edema [12]. Vasogenic edema formation results from a dramatic increase in BBB permeability and reflects an increase in T2-Weighted Resonance Imaging (T2WI) values [13]. Several studies have confirmed that elevated T2WI values are accompanied by decreased apparent diffusion coefficient (ADC) values, which reflect brain edema formation [13]. In addition, MRI may be potentially used as a stand to assess BBB permeability characteristics using small molecule paramagnetic contrast agents, such as gadolinium diethylene triamine pentaacetic acid (Gd-DTPA). The brain signal enhancement area is consistent with traditional markers labeled in the BBB-damaged region [14].

Currently, the molecular mechanism underlying treadmill pre-training-induced mitigation of cerebral edema mainly involves the effects of metalloproteinase (MMP) 9 and collagen IV on the BBB integrity [3], [8]. Accumulating evidence indicates that aquaporin 4 (AQP4), the most abundant water channel in the brain, also plays an essential role in the pathogenesis of cerebral edema [15].

AQP4 is found in high concentration in mammalian astrocytes, particularly in the periventricular region and subpial endfeet [16]. The transcription of AQP4–mRNA is increased specifically on Day 3 in the peri-infarcted cortex during a 7-day observation period after middle cerebral artery occlusion (MCAO) [17]. Genetic deletion of AQP4 ameliorates brain swelling following ischemia [18], while an accelerated progression of cytotoxic brain swelling was observed in AQP4 over-expressed mice [19]. Thus, regulation of AQP4 after cerebral ischemia is a potential target for the treatment of cerebral edema.

In this study, animals performed treadmill exercise for two weeks followed by transient middle cerebral artery occlusion (tMCAO). MRI was used to observe the cerebral edema development and the changes in BBB permeability. In addition, the expression of AQP4 was assessed using immunofluorescence and Western blotting analyses to explore the relationship between pre-exercise and cerebral edema.

## Materials and Methods

### Animals

This study was performed in strict accordance with the recommendations of the National Institutes of Health Guide for the Care and Use of Laboratory Animals. This protocol was approved by the Animal Experimental Committee of Fudan University, Shanghai, China (Permit Number: ETCA2013BN0002). All surgeries was performed using isoflurane anesthesia, and all efforts were made to minimize animal suffering.

All animals were housed under a 12-hour light/dark cycle with food and water provided ad libitum throughout the study. The animals were randomly divided into 3 groups: Sham, Treadmill pre-training (TT/Stroke) and Stroke.

### Experimental design

**Figure 1:**
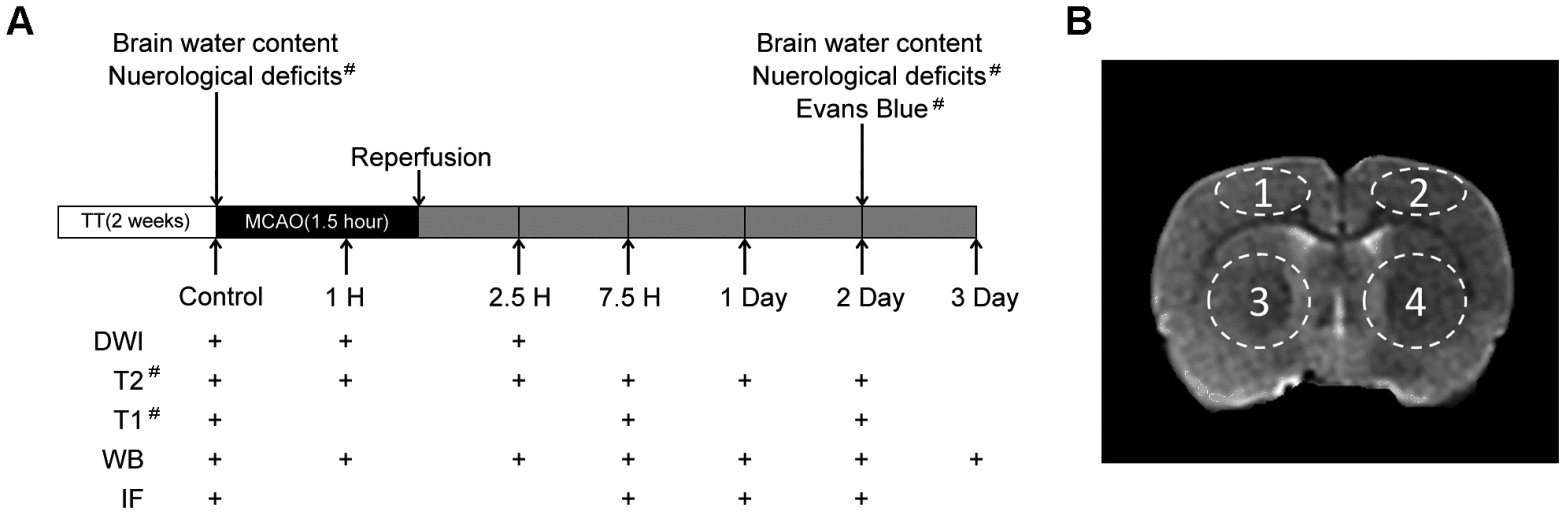
Experimental design and regions of interest. A, Experimental design: TT: treadmill pre-training; tMCAO: transient middle cerebral artery occlusion; Control: before tMCAO; WB: Western blotting; IF: immunofluorescence; +: experiment performed at that time point. #: Sham tMCAO performed. B, The regions of interest: 1: ipsilateral cortex; 2: contralateral cortex; 3: ipsilateral striatum; 4: contralateral striatum.

The experimental design is shown in figure 1A. A total of 180 animals were employed in the current study. 24 animals underwent MRI analysis, neurological deficit scoring and Evans Blue staining. 24 animals were analyzed for brain water content measurement. 48 animals were processed for immunofluorescence staining and 84 animals were processed for Western blotting analysed. Each group or each time point consisted of 6 animals and 137 rats completed the study.

### Treadmill pre-training

The treadmillpre-training protocol was performed according to our previous study [4]. Briefly, the rats began training at a speed of 5–8 m/minutes for 30 minutes each day for 2 days on a treadmill (DSPT-202 Type 5-Lane Treadmill; Litai Biotechnology Co., Ltd, China). Next, the speed increased to 20 m/minutes for 30 minutes daily for 5 days a week, which lasted for 2 weeks. The treadmill platform was kept level.

### Induction of cerebral ischemia

Experimental cerebral ischemia was induced by performing an occlusion of the middle cerebral artery (MCA) with some modifications as previously described [20]. Animals were anesthetized using 1% to 2% isoflurane, (Abbott, USA) in 70% N_2_O and 30% O_2_. The left common carotid artery (CCA), external carotid artery (ECA) and internal carotid artery (ICA) were exposed. The ECA was then ligated with a poly-L-lysine-coated intraluminal suture, which produced a larger infarction and reduces inter-animal variability to ensure the reliability of the stroke model. A flame-rounded tip with a 20 mm long surgical suture was introduced into the ICA to occlude the MCA. The suture was removed 90 minutes after the occlusion. Local cerebral blood reperfusion was detected using laser Doppler flowmetry. A minimum initial reduction of 80% in the laser Doppler reading after the MCA was occluded and a minimum restoration to 90% of the original baseline after reperfusion was considered a successful model of experimental stroke. The physiological parameters including blood pressure, heart rate, rectal temperature and blood gas were monitored before and after the surgeries. All the surgical procedures were performed in alternating order between the groups. After recoverying from anesthesia, The the rats were scored based on the basis of a 5-point scale [20]: 0, no neurological symptoms; 1, unable to completely extend the front jaw on the other side; 2, rotating while crawling, and falling to the other side; 3, unable to walk without help; and 4, unconsciousness. Rats with a score of 0 or 4 were excluded in from the study. We performed were performed the same surgical procedure, but without the MCA occluded occlusion in the sham group animals.

### MRI and data analysis

MRI was performed using a standard 3T clinical dedicated head MR scanner (Siemens Trio Tim, Germany) in which the gradients were achieved at 45 mT/m amplitude with a slew rate of 200 T/m/s per axis. The MR scanner was equipped with a dedicated four-channel phased array rat head coil assembly (Animal Coil 3T SIEMENS, Germany). Six rats per group were anesthetized with an intraperitoneal injection of 10% chloral hydrate at a dose of 400 mg/kg and their teeth and head were fixed in the prone position with the head on the coil central and coronal imaging. DWI-SE, TR = 3000, TE = 50 ms, field of view (FOV) = 40 mm, image matrix = 256×256 and at two different ‘b’ values (0, 1000 s/mm) were applied in three orthogonal directions. Trace-weighted apparent diffusion coefficient maps were generated.

T2WI-TSE images were obtained using the following parameters: TR = 3330 ms, TE = 68 ms, field of view (FOV) = 50 mm, image matrix = 256×256. For assessment of the brain water content and edema formation, T2-relaxometry measurement was performed at all time points (control, post-stroke 1-hour, 2.5-hours, 7.5-hours, 1-day, 2-day) using a Multi-Echo spin echo (SE) sequence with the following parameters: TR = 3330 ms, TE = 14.7/29.4/44.1/58.8/73.5/88.2 ms, FOV = 50 mm, image matrix = 192×192.

The T1-SE images were recorded before (pre-contrast) and after (post-contrast) administration of the contrast agent to assess the change in BBB permeability. Images were acquired with TR = 600 ms and TE = 12 ms, FOV = 58 mm, image matrix = 384×384. The post-contrast T1-SE was obtained 30 minutes after the administration of Gd-DTPA (MW: 590 Da, 0.2 mmol/kg, Magnevist, Shering, Germany).

All parameters were acquired from a single axial slice (1 mm thick) located at 7 mm posterior to the anterior tip of the frontal cortex as previously described [9]. The ADC and T2WI values were measured in the following 4 regions: ipsilateral striatum, contralateral striatum, and ipsilateral cortex and contralateral cortex as shown in figure 1B. In the striatal region, circular regions of interest were defined over the entire striatal region, excluding the ventricles (Figure 1.B). Volumetric estimations of the cerebral hemispheres were performed on the ADC and T2-weighted images using available built-in tools of the Siemens Syngo 2007A software (NUMARIS/4 syngo MR B15, Germany). The relative apparent diffusion coefficient (rADC) was calculated using the equation: rADC = (ipsilateral ADC/contralateral ADC)×100%. Analysis of the change in BBB permeability was performed on subtracted maps from the pre- and post-contrast T1-SE images to highlight the regions of Gd-DTPA extravasation. Gd-DTPA permeable BBB volume (PBV) in cubic centi-meters (cm^3^) represented brain tissue with a leaky BBB. The average pixel intensity (T1SI diff) of the hyper-intense enhanced regions derived from the subtraction maps were calculated using the built-in tools. The obtained values of the mean pixel intensity for the subtracted images (T1SI diff) were generated (SOMATOM syngo CT 2007A, Siemens AG, Munich, Germany). A product of the T1SI diff and PBV (T1SI diff×PBV) was considered to cause the observed temporal and spatial changes in the average pixel intensity of enhanced regions (T1SI diff) and the brain volume with leaky BBB (PBV). The T1SI diff×PBV product served as an indicator to quantify the overall entry of contrast agent into the brain over time.

### Neurological deficit scoring

Neurological deficit scoring was evaluated blindly using the modified Garcia score [21] after 2 days of ischemia. Five items were measured: spontaneous activity, symmetry of movements, outstretching while held by tail, reaction to touch on either side of the trunk and response to vibrissae touch. According to the degree of deficit, the animals were scored from 1 to 3, which represent poor to good responses, respectively, for each item.

### Sample collection

After MRI analysis and neurological deficit scoring, the animals were anesthetized using an intraperitoneal injection of 10% chloral hydrate at a dose of 400 mg/kg. After decapitation, the hemispheres were harvested for analysis.

### Brain water content determination

Wet weight (WW) and dry weight (DW) were used to evaluate the brain water content before tMCAO (n = 6) and 2 days after surgery (n = 6). The WW of the ischemic and contralateral hemisphere was separately recorded. The DW was recorded after drying the sample in an oven at 85°C for 72 hours. Brain edema was evaluated by measuring the water content using the formula of (WW-DW)/WW×100%.

### Determination of BBB permeability using Evans Blue staining

The permeability of the BBB was determined by measuring the amount of Evans blue (EB) dye (Sigma, St. Louis, MO, USA) 2 days after tMCAO. As previously described by Baskaya et al. [22], 4% of EB dye was administered intravenously at a dose of 4 ml/kg body weight and was allowed to circulate for 2 hours prior to measurement. The anesthetized animals were perfused transcardially with saline to wash away any remaining dye in the blood vessels prior to sample collection. Each hemisphere was weighed. The EB dye was extracted by homogenizing the sample in 3.5 ml of 0.1 mol/L phosphate buffered saline at pH 7.4. Proteins were precipitated by the addition of 6 ml of 60% trichloroacetic acid. The mixture was then vortexed for 2 minutes and cooled for 30 minutes. The sample was centrifuged for 40 minutes at 4000 rpm to pellet the brain tissue. Absorption of the supernatant was measured at a wavelength of 610 nm using a spectrophotometer (Eppendorf, US). The content of EB was measured as g/ml of brain tissue from each hemisphere using a standardized curve.

### Western blotting analysis

The isolated hemisphere was homogenized in a mixture of 250 mmol/L sucrose, 10 mmol/L Tris-HCl, pH 7.4, 0.2 mmol/L EDTA and 20 g/ml phenylmethyl-sulfonyl fluoride. Ten µg of soluble cortical and striatal proteins were resolved using 12.5% self-cast SDS-polyacryamide gel (SDS-PAGE) electrophoresis and subsequently transferred onto polyvinylidene difluoride (PVDF) membranes (Bio-Rad) m. The blot was then incubated overnight at 4°C with either a polyclonal antibody against AQP4 (1∶1000, Cell Signal Technology) or a monoclonal antibody against β-actin (1∶25,000, Sigma) in TBST (10 mmol/L Tris, pH 8.0,150 mmol/L NaCl, 0. 05% Tween 20) containing 3% skimmed milk. After washing, the membrane was incubated with horseradish peroxidase labeled antibody (1∶1000, Sigma) for 1 hour at room temperature. The membrane was incubated in ECL solution, and the gel image was captured using an imaging camera Bio-Rad and analyzed using gel image system (Quantity one) to estimate the integral optical density of the protein bands.

### Immunofluorescence staining and data analysis

The cerebral hemisphere was extracted after transcardial perfusion with 4% paraformaldehyde (solution in 0.1 mol/L phosphate buffer, pH = 7.4). The sample was then dehydrated in 20% sucrose solution in 4% paraformaldehyde, followed by 30% sucrose solution in 0.1 mol/L phosphate buffer. Non-specific blocking was performed on the sections of 1 hour with 10% goat serum at room temperature. Incubation with a polyclonal mouse anti-AQP4 (1∶1000, Cell Signal Technology) and a polyclonal rabbit anti-GFAP (1∶2000, Abcam) antibodies were subsequently performed at room temperature for 1 hour and then overnight at 4°C. The sections were then incubated in two fluorescence-coupled secondary antibodies (1∶1000, goat anti-mice Alexa-Fluor-488 nm and goat anti-rabbit Alexa-Fluor-555 nm, Abcam) for 1 hour at room temperature. For densitometric analysis, a computerized camera-based NIH Image-analysis system software (available at: http://rsb.info.nih.gov/nih-image/) was used as previously described [23]. Three sections (Bregma 1.0 mm) obtained from each animal were selected for analysis. Three randomly selected areas in the ischemic penumbra of each section were digitized into TIFF images using the same exposure time. The images were then binarized and segmented under a consistent threshold (50%). The total pixels per image field (300×300) were then measured. The average pixels of the ipsilateral and contralateral hemispheres were calculated for each animal. Tominimize the differences in the fluorescent intensity among the immunostained sections, the pixels values were presented as ratio of injury in the ipsilateral (IL) relative to the contralateral (CL) hemisphere (IL:CL = lesion: intact hemisphere).

### Statistical analysis

Statistical analysis was performed using SPSS for Windows, version 11.0 (SPSS Inc, Chicago, IL). The values were expressed as the mean ± s.e. (s.e.m.). One-way analysis of variance (ANOVA) followed by post hoc Fisher's LSD tests was used for within-group comparisons. Unpaired t-tests were applied for between-group comparisons of the brain water content, neurological deficits, Evans Blue, rADC, T2 values, T1Sidif×PBV, Western blotting analyses and immunofluorescence. A p-value of less than 0.05 was considered significant. Statistical charts were generated in the study using scientific software (Graphpad Prism Version 5.00 for Windows, Graph-pad Software, San Diego, CA, USA).

## Results

### Treadmill pre-training ameliorated brain edema after cerebral ischemia

**Figure 2:**
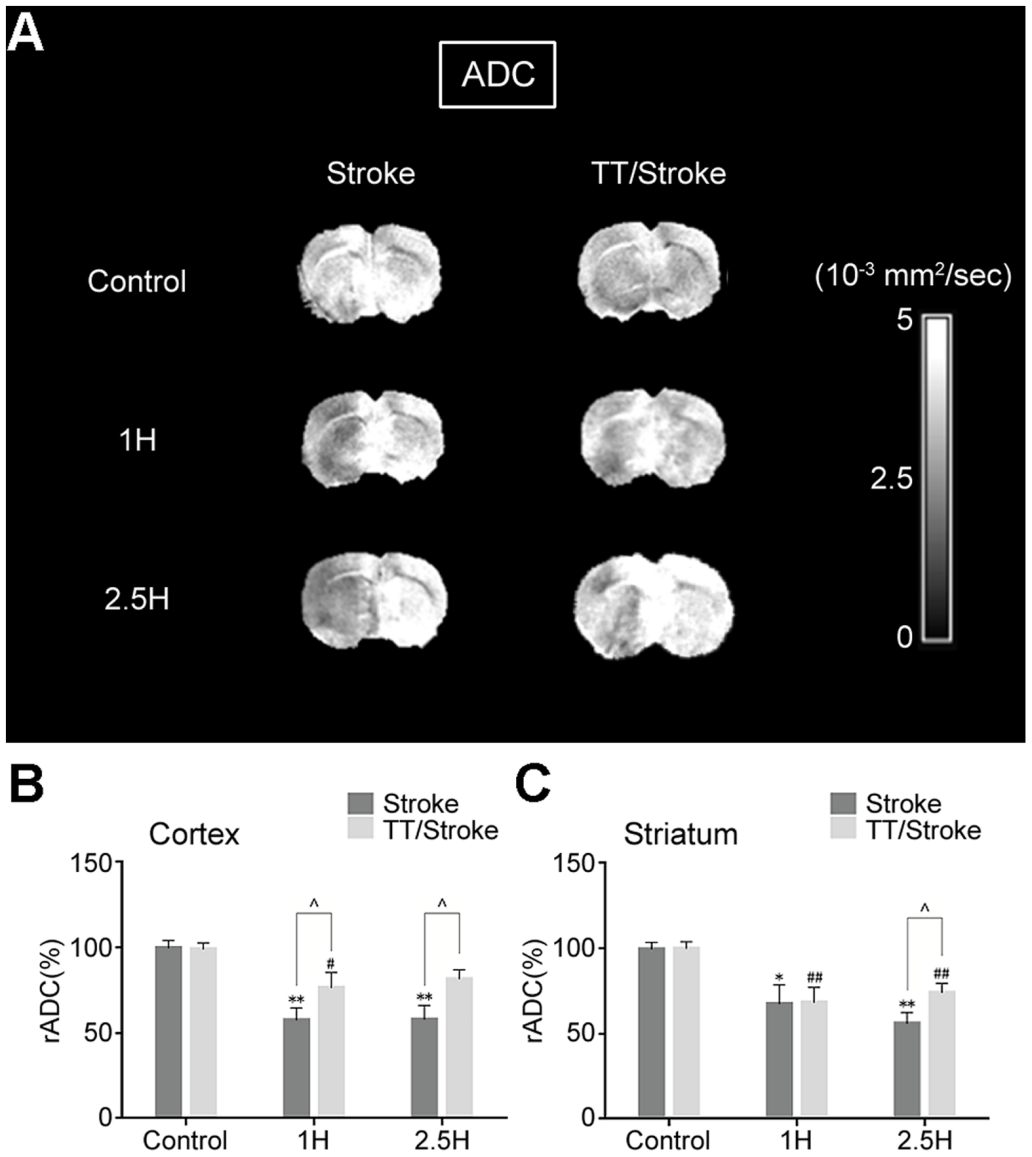
ADC images of rats in hyperacute cerebral infarction. MRI images (A), analysis in cortex region (B) and analysis in striatum region (C) of apparent diffusion coefficient (ADC) of Stroke and TT/Stroke groups in control, 1 hour and 2.5 hours after tMCAO. rADC: ipsilateral ADC/contralateral ADC×100%. * and **: significant difference when compared to the Control group and Stroke group (p<0.05) and (p<0.01) respectively; # and ##: significant difference when compared to the Control and TT groups (p<0.05) and (p<0.01) respectively; ∧: significant difference when compared to the TT/Stroke and Stroke groups at the same time point (p<0.05).

**Figure 3:**
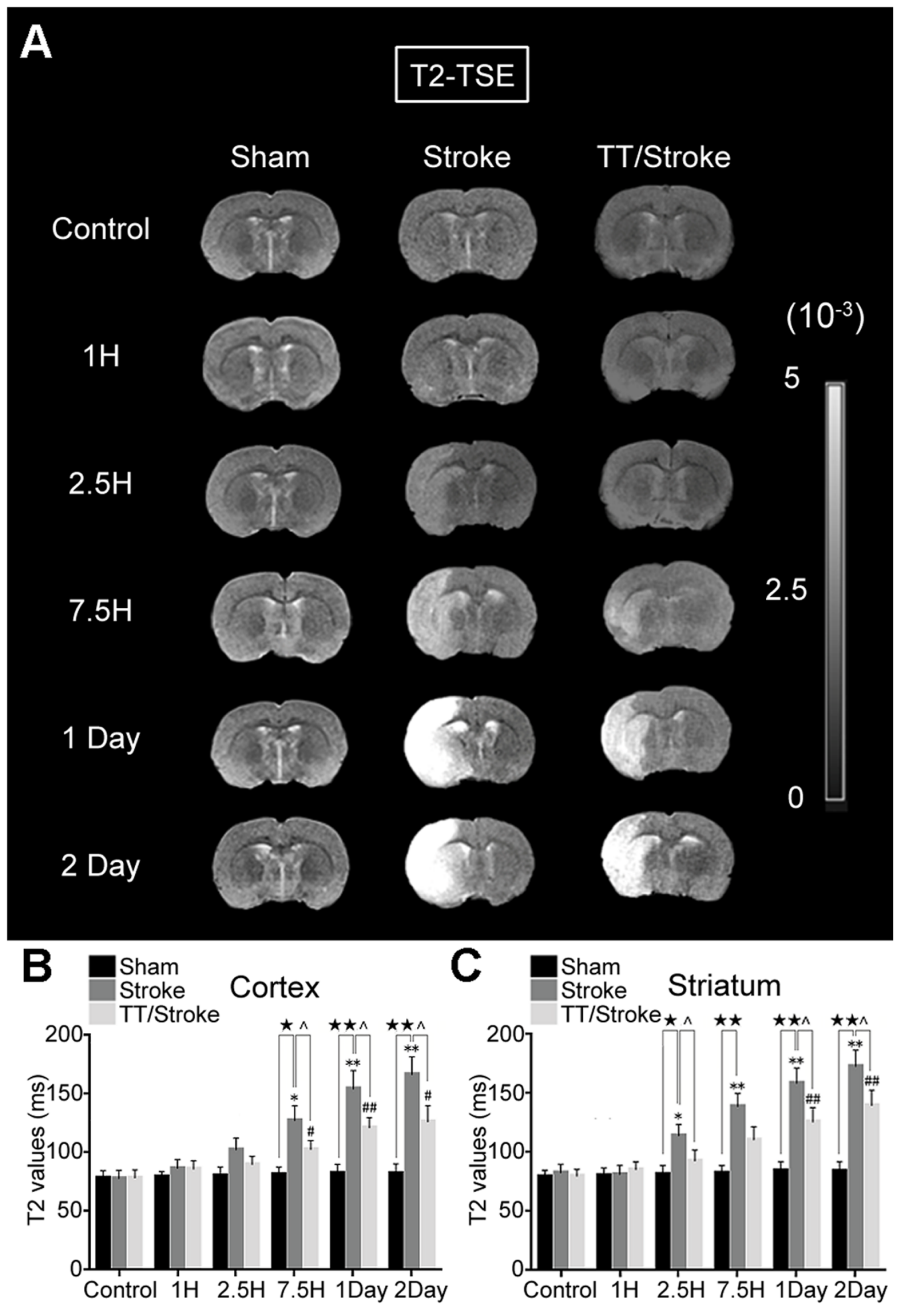
T2 images during cerebral ischemia and reperfusion injury. MRI images (A), analysis in the cortical region (B) and analysis in striatal region (C) of the T2-turbo spin echo (T2-TSE) of Sham, Stroke and TT/Stroke groups in control, 1 hour, 2.5 hours, 7.5 hours, 1 day and 2 days after tMCAO. * and **: significant difference when compared to the Control and Stroke groups (p<0.05) and (p<0.01) respectively; # and ##: significant difference when compared Control group and TT group (p<0.05) and (p<0.01) respectively; ★ and ★★: significant difference when compared to the Stroke and Sham groups at the same time point, (p<0.05) and (p<0.01) respectively; ∧: significant difference when compared to the TT/Stroke and Stroke groups at the same time point (p<0.05) respectively.

In hyperacute cerebral infarction (1 h and 2.5 h after experimental stroke), the rADC of the ipsilateral hemisphere of both TT/Stroke group and Stroke group decreased after ischemia and showed a similar pattern in the cortex and striatum. Treadmill pre-training could improve the decrease in rADC when compared with the Stroke only group. In the cortex, the rADC of the TT/Stroke group significantly decreased less at 1 hour and 2.5 hours after ischemia by 33.0% and 39.5%, respectively (vs. Stroke group, p<0.05) (Figure 2B). In the striatum, treadmill pre-training decreased the rADC value less by 28.9% compared with the Stroke group (p<0.05) at 2.5 hours after ischemia (Figure 2C). The T2 values of the ipsilateral hemisphere were increased after ischemia in the two groups and were altered in a similar pattern in the ipsilateral cortex and striatum. In the cortex, T2 values of the TT/Stroke group increased less when compared with the Stroke group (7.5-hour by 20.1%, 1-day by 21.5%, 2-day by 23.2%) (p<0.05) (Figure 3B). In the striatum, T2 values of the TT/Stroke group increased less compared with the Stroke group (2.5-hour by 18.1%, 1-day by 19.8%, 2-day by 19.5%) (p<0.05) (Figure 3C). These results showed that treadmill pre-training could down-regulate the increased T2 values after ischemia.

### Treadmill pre-training improved BBB integrity

**Figure 4:**
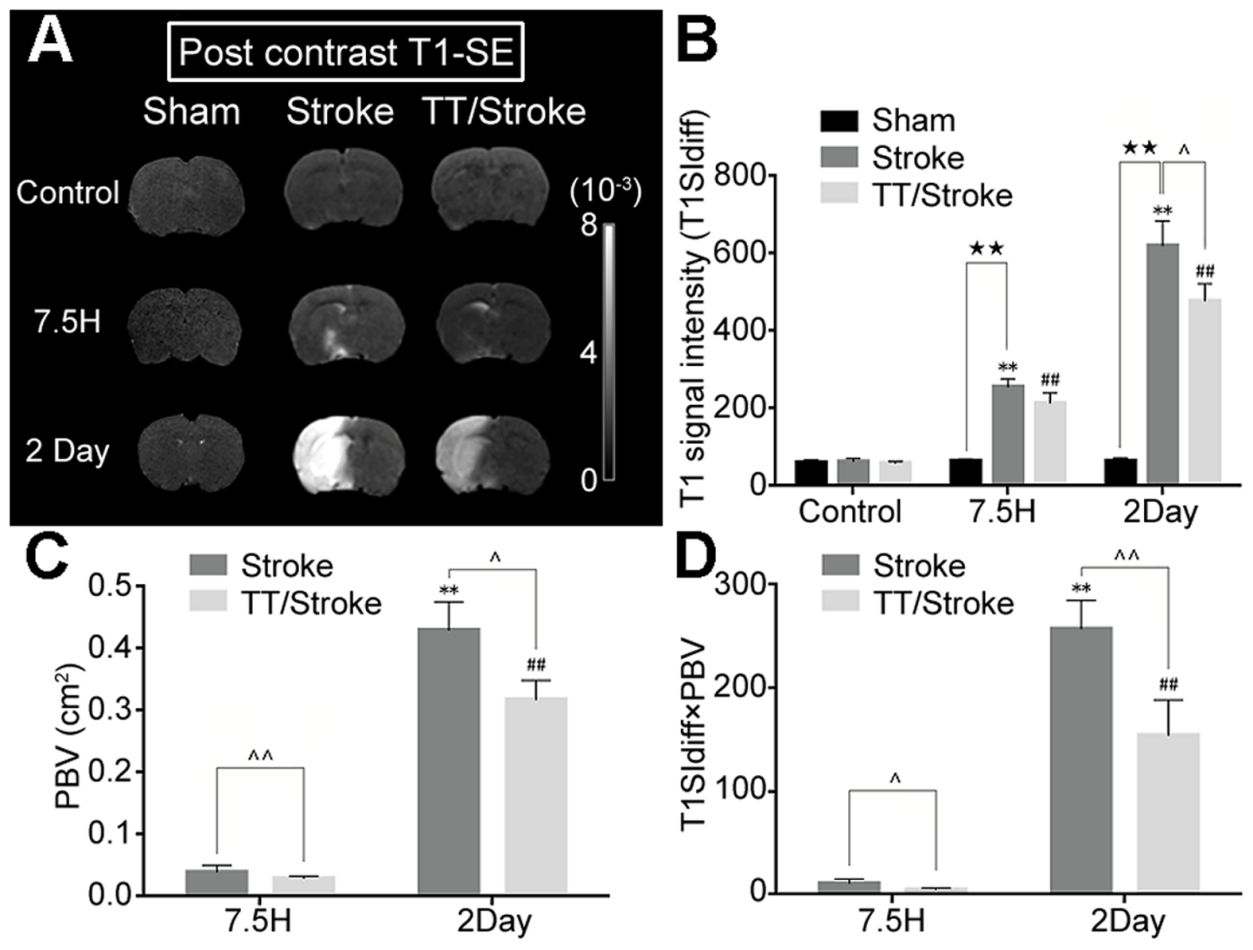
Post-contrast T1 images after experimental stroke. MRI images (A), analysis of T1 signal intensity (T1SI diff) (B), analysis of permeable BBB volume (PBV) (C) and analysis of T1SI diff×PBV (D) of post contrast T1-spin echo (T1-SE) of Sham, Stroke and TT/Stroke groups in control, 7.5 hours and 2 days after tMCAO. **: significant difference when compared Control group and Stroke group (p<0.01); ##: significant difference when compared Control group and TT group (p<0.01); ★★: Significant difference when compared Stroke group and Sham group at the same time point (p<0.01); ∧ and ∧∧: significant difference when compared TT/Stroke group and Stroke group at the same time point (p<0.05) and (p<0.01) respectively.

**Figure 5:**
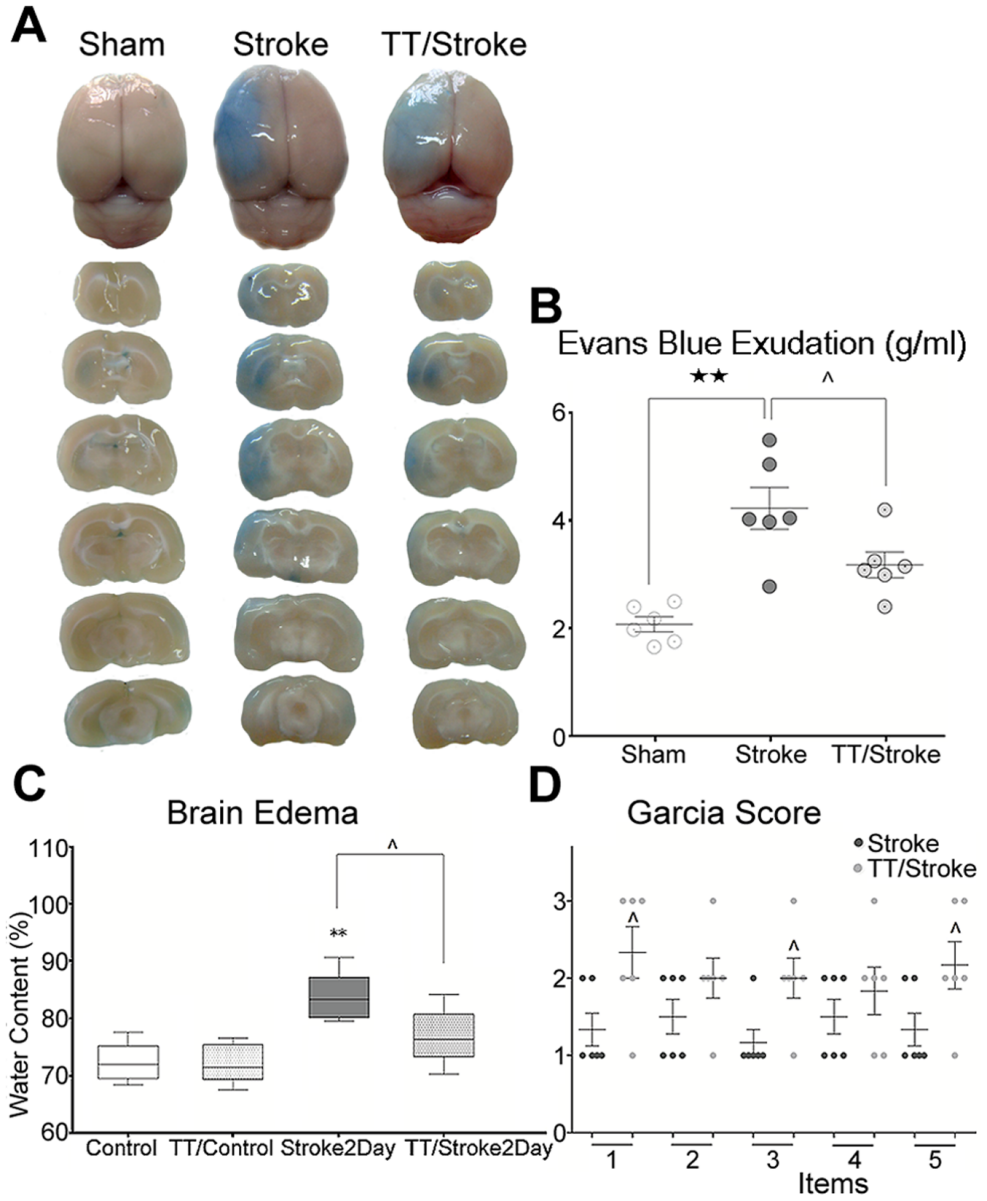
Evans Blue assay, brain water content and Garcia score. Evans Blue images (A), analysis of Evans Blue exudation (g/ml of brain tissue)(B) of Sham, Stroke and TT/Stroke groups at 2 days after tMCAO. Brain water content before (control group and TT/control group) and 2 days after cerebral ischemia (C), measured by (wet weight – dry weight)/wet weight*100%. Analyses of Garcia score (D) of Stroke and TT/Stroke groups at 2 days after tMCAO. The Sham group had no deficit in Garcia score. Items: 1, spontaneous activity; 2, symmetry of movements; 3, outstretching while held by tail; 4, reaction to touch on either side of trunk; 5, response to vibrissae touch. **: significant difference when compared Control group and Stroke group (p<0.01). ★★: significant difference when compared Stroke group and Sham group at the same time point (p<0.01); ∧: significant difference when compared TT/Stroke group and Stroke group at the same time point (p<0.05) respectively. Control: before tMCAO. Sham: sham tMCAO.

The post-contrast T1 images showed that T1SIdiff of the ipsilateral hemisphere increased after ischemia. Treadmill pre-training down-regulated the increase of the T1SIdiff value by 24.2% when compared with the Stroke group at 2 days after ischemia (Figure 4B). The PBV at 2 days significantly increased when compared with the PBV at 7.5 hours after ischemia in the TT/Stroke and Stroke only groups (p<0.01). Treadmill pre-training decreased the PBV value by 50.0% at 7.5 hours (vs. Stroke group, p<0.01) and 25.5% at 2 days (vs. Stroke group, p<0.05) after ischemia (Figure 4C). For the product of T1SIdiff×PBV, treadmill pre-training decreased the product by 59.7% at 7.5 hours (vs. Stroke group, p<0.05) and by 25.5% at 2 days (vs. Stroke group, p<0.05) (Figure 4D). EB exudation of the ipsilateral hemisphere also significantly increased when compared to the Sham group at 2 days after ischemia (p<0.01, Figure 5B). In addition, treadmill pre-training could also significantly down-regulate the EB exudation when compared to the Stroke group (p<0.05). Simultaneously, we also detected the water content of ipsilateral cerebral hemisphere in both groups. The water content of ipsilateral cerebral hemisphere increased at 2 days after ischemia compared with the control (p<0.01) (Figure 5C). A significant decrease was observed in the TT/Stroke group at 2 days after tMCAO (p<0.05) (Figure 5C), which also supported our results.

### Treadmill pre-training mitigates the neurological deficits

The Garcia score at 2 days after cerebral ischemia is shown in figure 5D. The neurological deficit was significantly reduced in the items of spontaneous activity, outstretching while held by tail and response to vibrissae touch after treadmill pre-training (p<0.05).

### Treadmill pre-training down-regulated the expression of AQP4

**Figure 6:**
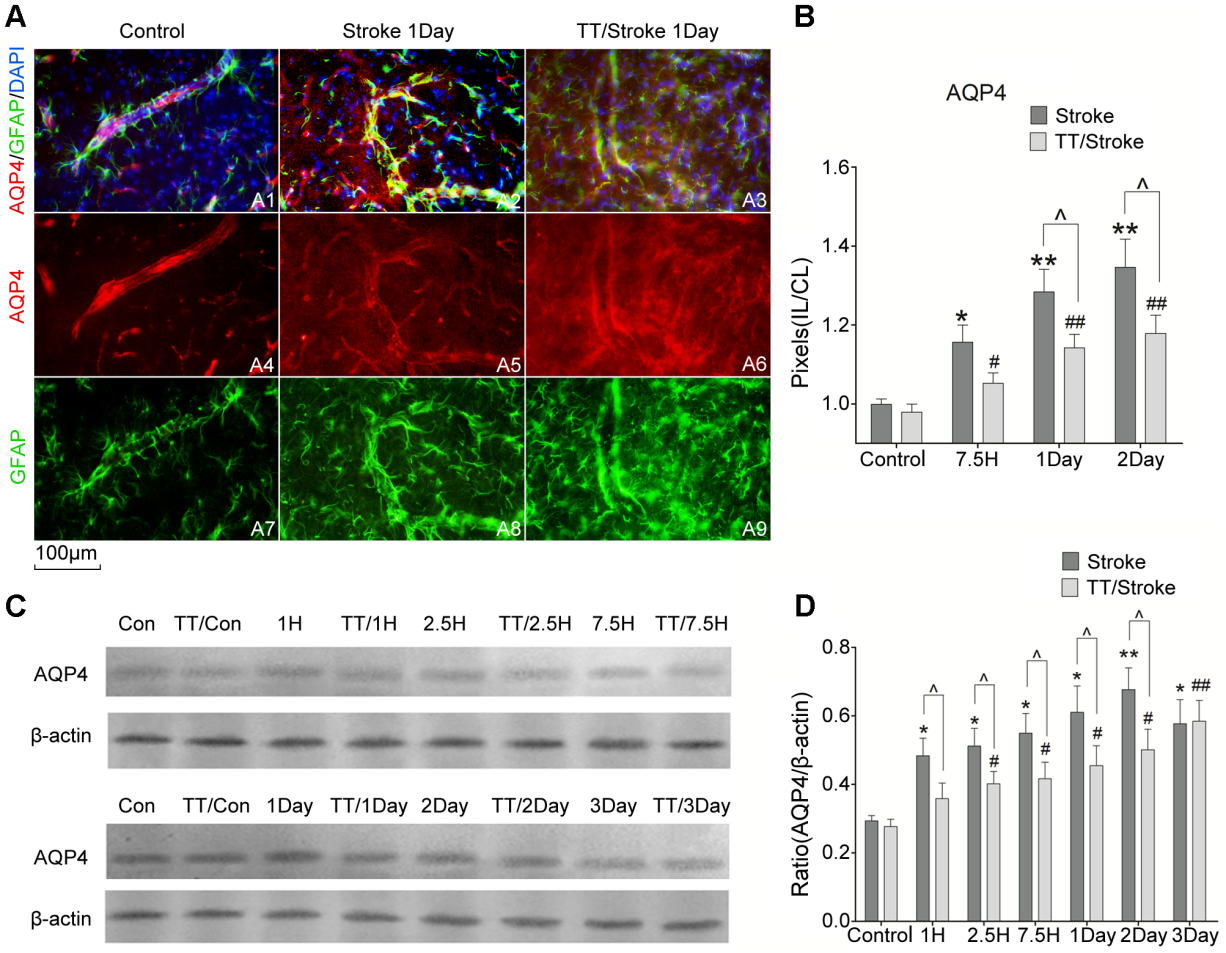
Immunofluorescence and Western Blot. The image (scale bar: 100 µm) of immunofluorescence (A) of co-expression of AQP4 and GFAP (A1–A3), expression of AQP4 (A4–A6) and expression GFAP (A7–A9) of the cerebral microvascular of ischemic cortex of TT/Stroke and Stroke groups 1 day after tMCAO. Analysis of pixels (IL/CL) of AQP4 of TT/Stroke and Stroke groups in control, 7.5 hours, 1 day and 2 days after tMCAO (B). AQP4: Aquaporin-4, GFAP: Glial fibrillary acidic protein, IL: ipsilateral, CL: contralateral. Western blots image (C) and quantitative analysis of ratio (AQP4/β-actin) of the longitudinal expression of AQP4 and β-actin of TT/Stroke and Stroke groups from control to 3 days after tMCAO (D). * and ** : p<0.05 and p<0.01 respectively when compared Stroke group and control group; # and ## : p<0.05 and p<0.01 when compared TT/Stroke group and control group; ∧ : p<0.05 when compared TT/Stroke group and Stroke group at the same time point.

Immunofluorescent staining in the cortical region around ischemic lesions showed that AQP4 was mainly expressed in astrocytic foot processes labeled by glial fibrillary acidic port (GFAP) around the brain microvascular tissue (Figure 6A4 to 6A6). The number of AQP4-positive cells increased gradually from 7.5 hours to 2 days after ischemia. A significant decrease in AQP4-postive cells number was observed in the TT/Stroke group at 1 day and 2 days after ischemia when compared with the Stroke group (p<0.05) (Figure 6B). In figure 6D, a gradual increase in the expression of AQP4 was observed in both the TT/Stroke and Stroke groups from 1 hour to 3 days after ischemia. A significant reduction in the expression of AQP4 was demonstrated in the TT/Stroke group when compared with stroke group at 1 hour, 2.5 hours, 7.5 hours,1 day and 2 days after ischemia (p<0.05).

## Discussion

This current study used MRI to observe the effectiveness of treadmill pre-training on the reduction of brain edema and protection of blood-brain barrier from damage after ischemia and reperfusion. Wet and dry weight measurements and the Evans Blue assay were used to support these findings. In addition, Western blotting analyses and immunofluorescence were used to confirm that treadmill pre-training could decrease the expression of AQP4during ischemia and reperfusion and to determine the relationship between treadmill pre-training and cerebral edema. Finally, the neurobehavioral score illustrated that the treadmill pre-training could improve motor function after ischemic injury.

MRI scan demonstrated that the rADC of the two groups (stroke group and TT/stroke group) began to decrease at 1 hour after ischemia, and there were statistical differences in the cortex and striatum, which might be due to the restriction of post-ischemic energy metabolism and Na^+^, K^+^-ATPase on the cell membrane activity. Once cytotoxic edema formed, the swelling of brain cells may have cause a restriction of the water molecules by diffusion and thus, a decrease in the apparent diffusion coefficient. At 2.5 hours after ischemia, the rADC of the cortex showed no further decline, while the decrease was maintained in the striatum of the Stroke group, although this difference was not statistical significance. For the TT/Stroke group, the rADC at 2.5 hours after ischemia showed no further decline except a slightly insignificant increasing pattern in both the cortex and striatum. This finding illustrates that treadmill pre-training can reduce water diffusion limitation and alleviate cytotoxic edema in cerebral ischemia. The different pattern in rADC between the cortex and striatum might be due to the different degree of injury at different brain regions. Several studies have demonstrated that the brain striatum is more vulnerable to ischemia injury in the current MCAO animal model [24].

The incidence of high-signal foci was observed grossly at 2.5 hours after ischemia (3 out of 6 in the Stroke group, 1 out of 6 in the TT/Stroke group). Statistical analyses showed that the T2 values of cortex and striatum in all animals gradually increased over time after ischemia. The T2 values increased at 2.5 hours after ischemia in the Stroke group; however, the increase was later in the TT/Stroke group. Treadmill pre-training could decrease the elevated T2 values after ischemia, indicating that treadmill pre-training can inhibit post-ischemic vasogenic edema and mitigate cerebral edema. The detection of brain water content also confirmed that treadmill pre-training could reduce edema after 2 days of ischemia. This observation was consistent with the results observed after 2 days of ischemia using T2 sequences.

Vasogenic edema stems from the BBB opening and previous animal studies [25] have reported a biphasic BBB opening after ischemia. The first opening was observed at 3 to 5 hours after ischemia and the second broader opening occurred at 48 hours after ischemia. Recent studies [9] have confirmed this phenomenon using MRI scanning and observed that at 24 hours after reperfusion, the BBB permeability significantly decreased when compared with 4 hours of reperfusion. To examine the effect of treadmill pre-training on BBB permeability, the Ga-DTPA contrast agent was employed. The TT/Stroke group showed a decrease in the semi-quantitative amount of contrast agent leakage (T1SIdiff×PBV) when compared with the Stroke group at 7.5 hours and 2days after ischemia. Thus, it reduces BBB damage after ischemia/reperfusion. Previous studies [8] also demonstrated that pre-training protected BBB integrity using the EB assay via the reduction in MMP-9 expression. This finding is consistent with the findings obtained in the present study, where the MRI provides an advantage in observing the biological event in a live and dynamic manner.

Aquaporin-4 (AQP4) is a hydro-selective membrane transport protein which is expressed in glial cells of the brain particularly at the borders between the brain parenchyma and major fluid compartments. Strong AQP4 expression was found in astroglial cell foot processes at the BBB, in the glia lining of the subarachnoid cerebrospinal fluid space and in the ependyma and subependymal glia lining of the ventricular cerebrospinal fluid space [26], [27]. AQP4 plays a key role in brain water balance in central plasma osmolarity and cerebral edema formation and regression in pathological disease [28]. AQP4 immunoreactivity around the ischemic foci was also significantly stronger than the central area, and its expression increased with brain edema formation in human autopsy [29]. Mice deficient in AQP4 suffer less brain edema after an acute water intoxication and ischemic stroke [18]. Transgenic mice lacking the dystrophin-associated protein complex in which end-feet pooled AQP4 are anchored, showed less severe ischemic edema compared to wild-type mice [30]. Genetic deletion of a syntrophin in which a member of the dystrophin-associated protein complex is also reduced in ischemic edema [31], [32]. Previous studies have also found that an injection of AQP4 inhibitors,2-(Nicotinamide)-1,3,4-thiadiazole (TGN-020), couldameliorate cerebral edema in the mouse brain at 24 hours after arterial ischemia [33]. In contrast, AQP4 overexpression in transgenic mice accelerated the progression of brain edema [19]. Taken together, these findings indicate that AQP4 is essential to control cerebral water balance and plays an important role in BBB permeability.

In this study, immunofluorescence techniques provided morphological evidence that treadmill pre-training lowers the level of AQP4 expression at the cortical border zone. Frydenlund et al. [34] utilized quantitative immunogold cytochemistry to observe changes in the expression of AQP4 in the ischemic cortex and striatum. The striatal core displayed a loss in perivascular AQP4 at 24 hours of reperfusion with no sign of subsequent recovery. The most affected part of the cortex also exhibited a loss in perivascular AQP4 but demonstrated a partial recovery at 72 hours of reperfusion. Their study also revealed that the cortical border zone differed from the central part of the ischemic lesion by showing no loss of perivascular AQP4 at 24 hours of reperfusion rather than a slight increase. This finding was consistent with the immunofluorescent results obtained in this study. In addition, on the basis of the Western blotting results, it can be estimated that mislocalization of AQP4 anchoring at the perivascular membrane after cerebral ischemia rather than a net loss of AQP4, results in a disruption of AQP4, which was quantified using immuno-electron microscopy and the minor difference in Western blotting analyses. In our study, we detected changes in AQP4 expression change using Western blotting analyses. These results showed that AQP4 protein expression was enhanced after ischemia at 1 hour and it increased with time and peaked at 2 days after ischemia. Treadmill pre-training could significantly reduce the expression of AQP4 from 1 hour to 2 days after ischemia. However, it is still unclear if treadmill pre-training can change the polarization of AQP4 expression.

However, there are still reported conflicts regarding the role of AQP4 in ischemic stroke. AQP4-knockout mice suffer from vasogenic brain edema [35], [36]. Saadoun et al. found that deletion of AQP4 in mice does not produce major structural abnormalities in the brain [37]. Moreover, Bonomini and Rezzani reported the relationship between aquaporin and BBB and suggested that AQP4 deficiency may reduce cytotoxic edema, but increase vasogenic edema [38]. In our study, we found that treadmill pre-training mitigated brain edema and BBB disruption, but down-regulated the expression of AQP4 after reperfusion, which indicated that the neuroprotective effects of exercise may be correlated with down-regulation of AQP4. Interestingly, on the basis of the Western blot result at 3 days after ischemia, the expression of AQP4 in the TT/Stroke group was nearly similar to the Stroke group., The long-term effects of AQP4 expression after cerebral ischemia regulated by exercise preconditioning remains unclear and should be studied further.

Our previous studies have shown that treadmill pre-training can decrease the concentration of extracellular glutamate [4], [5]. This phenomenon may be related to the up-regulation of gamma-aminobutyric acid (GABA) and changes in the expression of glutamate transporter 1 (GLT-1). However, other studies have shown that GLT-1 and AQP4 are widely co-expressed in astrocytes and GLT-1 is characteristic of water transport [39]. Down-regulation of astrocytic GLT-1 expression was observed in AQP4 knockout mice and may reduce the uptake of glutamate [39].

Since exercise is a systemic treatment and exerts regional protection in the brain, the presence of a bridge from system to region can be confirmed. A previous study found that changes in blood-borne factors contributed to the decline in neurogenesis and cognitive impairments during aging [40]. It can also be hypothesized that treadmill pre-training may change the level of some factors in the blood and thus, generate neuroprotective effects on the central nervous system via the vasculature. However, there is no evidence to suggest any specific therapeutic targets strongly correlating with treadmill pre-training, and the mechanism of cerebral ischemia tolerance or neuroprotection induced by treadmill pre-training remains unclear. In the present study, we found that treadmill pre-training down-regulated AQP4 expression, ameliorates brain edema and maintains the integrity of BBB. Previous studies have shown that interleukin-1β (IL-1β) and Tumor Necrosis Factor-α (TNF-α) have the ability to up-regulate the expression of AQP4 [41], [42]. Both IL-1β and TNF-α are closely involved in neuroinflammation. Ding et al. have demonstrated that chronically increased expression of TNF-alpha during exercise prevented the acute elevation of TNF-alpha expression, which ameliorated inflammatory injury after cerebral ischemia/reperfusion [43]. Moreover, pre-existing systemic inflammation increased the levels of IL-1 in the ischemic cerebral cortex [44], which also suggested that AQP4 may be a potential target for systemic inflammation, result in increased brain edema. Taken together, the down-regulation of AQP4 expression after stoke may be correlated with pre-existing systemic inflammation, which results in regional changes in inflammatory factors in the brain during treadmill pre-training. Furthermore, there are evidences that have shown that exercise may induce angiogenesis and improve cerebral blood flow [45], [46]. These neuroprotective effects may be caused by enhanced collateral circulation in the ischemic core and penumbra area. Thus, it is interesting to further investigate whether treadmill pre-training improves regional blood supply or brain ischemia tolerance.

### Conclusion

From the results of AQP4 protein studies and brain edema development with MRI, the treadmill pre-training could minimize the ischemic brain edema formation and the down-regulate of AQP4 expression and this is consistent with the other studies [47], [48]. Therefore, pre-conditioning training might act as an effective intervention to reduce the degree of ischemia-induced edema by lowering AQP4 expression. This study demonstrated that treadmill pre-training can delay or ameliorate the development of ischemic brain edema and it may be achieved by down-regulating the expression of AQP4. As a safe and effective preventive neuroprotection strategy, exercise serves as a potential pre-treatment for people, especially those with high risk factors, to reduce the risk and improve the outcome of stroke.
